# Using Genetic Variation and Environmental Risk Factor Data to Identify Individuals at High Risk for Age-Related Macular Degeneration

**DOI:** 10.1371/journal.pone.0017784

**Published:** 2011-03-24

**Authors:** Kylee L. Spencer, Lana M. Olson, Nathalie Schnetz-Boutaud, Paul Gallins, Anita Agarwal, Alessandro Iannaccone, Stephen B. Kritchevsky, Melissa Garcia, Michael A. Nalls, Anne B. Newman, William K. Scott, Margaret A. Pericak-Vance, Jonathan L. Haines

**Affiliations:** 1 Center for Human Genetics Research, Vanderbilt University, Nashville, Tennessee, United States of America; 2 John P. Hussman Institute for Human Genomics, Miller School of Medicine, University of Miami, Miami, Florida, United States of America; 3 Vanderbilt Eye Institute, Vanderbilt University Medical Center, Nashville, Tennessee, United States of America; 4 Hamilton Eye Institute, University of Tennessee Health Science Center, Memphis, Tennessee, United States of America; 5 Department of Preventive Medicine, University of Tennessee Health Science Center, Memphis, Tennessee, United States of America; 6 Sticht Center on Aging, Wake Forest University, Winston-Salem, North Carolina, United States of America; 7 Laboratory for Epidemiology, Demography, and Biometry, National Institute on Aging, Bethesda, Maryland, United States of America; 8 Laboratory of Neurogenetics, National Institute on Aging, Bethesda, Maryland, United States of America; 9 Department of Epidemiology, University of Pittsburgh, Pittsburgh, Pennsylvania; Ohio State University Medical Center, United States of America

## Abstract

A major goal of personalized medicine is to pre-symptomatically identify individuals at high risk for disease using knowledge of each individual's particular genetic profile and constellation of environmental risk factors. With the identification of several well-replicated risk factors for age-related macular degeneration (AMD), the leading cause of legal blindness in older adults, this previously unreachable goal is beginning to seem less elusive. However, recently developed algorithms have either been much less accurate than expected, given the strong effects of the identified risk factors, or have not been applied to independent datasets, leaving unknown how well they would perform in the population at large. We sought to increase accuracy by using novel modeling strategies, including multifactor dimensionality reduction (MDR) and grammatical evolution of neural networks (GENN), in addition to the traditional logistic regression approach. Furthermore, we rigorously designed and tested our models in three distinct datasets: a Vanderbilt-Miami (VM) clinic-based case-control dataset, a VM family dataset, and the population-based Age-related Maculopathy Ancillary (ARMA) Study cohort. Using a consensus approach to combine the results from logistic regression and GENN models, our algorithm was successful in differentiating between high- and low-risk groups (sensitivity 77.0%, specificity 74.1%). In the ARMA cohort, the positive and negative predictive values were 63.3% and 70.7%, respectively. We expect that future efforts to refine this algorithm by increasing the sample size available for model building, including novel susceptibility factors as they are discovered, and by calibrating the model for diverse populations will improve accuracy.

## Introduction

Age-related macular degeneration (AMD) attacks the central retina and causes debilitating vision loss in the approximately 1.5 million Americans affected by advanced forms of disease[1].Genetic variants CFH Y402H[2]–[4], ARMS2 A69S[5], [6], C3 R102G[7]–[9], and cigarette smoking[10] are now well-accepted risk factors for AMD, and CFB R32Q is associated with decreased AMD risk[11]–[13]. This recent success in identifying both genetic and environmental modifiers of AMD susceptibility has prompted the development of algorithms for identifying individuals at particularly high risk for AMD based on some combination of environmental and genetic risk factor data[11], [14]–[18] (Table 1). Comparisons between algorithms have been made difficult due to differences in which risk factors are included in the model, differences in the type of modeling strategy used, and the variety of measures used to describe the success of the algorithms. However, it is obvious that no current algorithm correctly classifies AMD case-control status 100% of the time.

**Table 1:** Previous studies that developed an AMD algorithm.

Reference	Factors in the Model	Method(s) Used	Independent Dataset for Validation?	Sensitivity	Specificity	AUC
Gold et al. 2006[11]	CFH, C2, CFB	Genetic Algorithm	yes	0.74	0.56	.
Hughes et al. 2007[14]	CFH, ARMS2, smoking	risk score	no	.	.	.
Jakobsdottir et al. 2008[15]	CFH, ARMS2, C2, CFB	logistic regression	no	.	.	.
Jakobsdottir et al. 2008[15]	CFH, ARMS2, C2, age, gender, smoking	Generalized MDR	no	0.70	0.74	.
Jakobsdottir et al. 2009[16]	CFH, ARMS2, C2	logistic regression	no	.	.	0.79
Seddon et al. 2009[17]	CFH, ARMS2, C2, CFB, C3, CFH*supplement treatment group, age, gender, education, baseline AMD grade, smoking, BMI	logistic regression	no	.	.	0.83
Gibson et al. 2010[18]	CFH, ARMS2, C3, SERPING1, age, gender, smoking	logistic regression	no	0.76	0.76	0.83

The level of accuracy needed to reach clinical utility is debatable and depends on a variety of factors, including whether the underlying goal is presymptomatic diagnosis or screening for increased risk of future disease. The receiver operating characteristic (ROC) curve plots sensitivity vs. 1-specificity and can be used to evaluate how well a continuous variable (e.g. probability of a genetic disease calculated from a genetic algorithm) can discriminate between binary outcomes (e.g. case-control status). A common rule of thumb for evaluating clinical tests is that the area under the ROC curve (AUC) should be >0.75 for screening individuals at increased risk for disease and >0.99 for presymptomatic diagnosis[19]. Of the three previous studies reporting the AUC in AMD, all exceeded the threshold set for screening[16]–[18], though the authors were cautious in raising the possibility that an accurate genetic test for AMD could be developed. An absolutely critical step for gauging the clinical utility of any algorithm is to determine its accuracy in a completely untested dataset of individuals at risk for AMD. This step mimics the situation that would occur should an algorithm begin to be used in clinical practice, and the importance of performing this validation step cannot be overstated. To our knowledge, none of these studies tested their models in an independent dataset. The single study that did have separate training and testing datasets reported 70% sensitivity and 50% specificity in their testing dataset[11]. While this represents unprecedented success in modeling a complex genetic disease and would potentially be useful in identifying high-risk persons, a specificity of 50% makes a “low-risk” result difficult to interpret.

Therefore, in developing a new algorithm, our goals were twofold: 1) to increase the accuracy to a level approaching clinical usefulness, and 2) to carry out a true test of model validation by thoroughly testing the new algorithm in an independent, population-based dataset. We chose to model the effects of age, smoking, CFH Y402H, ARMS2 A69S, C3 R102G, and CFB R32Q using logistic regression, multifactor dimensionality reduction (MDR), and grammatical evolution of neural networks (GENN) in multiple distinct datasets. First, we constructed these models in 4/5 of the Vanderbilt-Miami (VM) case-control study population and then tested the models on the remaining 1/5 of this dataset and another VM dataset of families containing multiple affected individuals. For a more rigorous test of how the models would perform in the population as a whole, we then applied them to the Age-Related Maculopathy Ancillary (ARMA) Study, which was drawn primarily from the Health ABC cohort, a population-based longitudinal study of highly functional elderly individuals randomly selected from Medicare roles in Memphis, TN. Among other measures, overall correct classification rate, AUC, positive predictive value (PPV), and negative predictive value (NPV) were used to evaluate the success of the models in this final testing set.

## Methods

### Ethics Statement

Approval for the study was obtained from Institutional Review Boards at VanderbiltUniversity, University of Miami, and University of Tennessee Health Science Center. All study participants gave written informed consent to participate in this study, and this research adhered to the tenets of the Declaration of Helsinki.

### Study Populations

It is essential to construct a model in one dataset and then apply the model in a separate dataset to avoid bias in model evaluation. With this in mind, we initially used one dataset for training (referred to as the Vanderbilt-Miami (VM) training dataset), and three independent datasets for testing (the VM testing dataset, the VM family dataset, and the ARMA Study). Later, the analyses were reversed with the ARMA dataset being used for training and the VM datasets used for testing.

The VM training dataset was formed by randomly selecting 4/5 of the AMD cases (n = 349) and 4/5 of the controls (n = 216) with complete risk factor data, who were ascertained through ophthalmology clinics at Vanderbilt and Duke University Medical Centers. The remaining 1/5 cases (n = 87) and 1/5 controls (n = 54) were assigned to the VM testing dataset. The VM family dataset was used only for testing the models and consisted of 226 families with multiple affected individuals and their unaffected relatives. There was no overlap of individuals between the VM training, testing, and family datasets. Individuals not of European descent (n = 33) were excluded from analysis due to the small sample size and because allele frequencies of some AMD-associated variants differ by ancestry[20]. All patients and controls received an eye exam and had stereoscopic fundus photographs graded according to a modified version of the Age-Related Eye Disease Study (AREDS) grading system as described elsewhere [21], [22]. Briefly, grades 1 and 2 represent controls. Grade 1 controls have no evidence of drusen or small non-extensive drusen without pigmentary abnormalities, while grade 2 controls may show signs of either extensive small drusen or non-extensive intermediate drusen and/or pigmentary abnormalities. Grade 3 AMD cases have extensive intermediate drusen or large, soft drusen with or without drusenoid retinal pigment epithelial detachment. Grade 4 AMD cases exhibit geographic atrophy and grade 5 individuals have exudative AMD, which includes nondrusenoid retinal pigment epithelial detachment, choroidal neovascularization, and subretinal hemorrhage or disciform scarring. Individuals were classified according to status in the more severely affected eye.

The ARMA samples (n = 85 cases, 148 controls) were part of a prospective cohort from Memphis, TN, aged 70 or older. The vast majority of participants (86%) were from the Memphis Health ABC study, which included individuals who did not have difficulty walking a quarter of a mile or climbing a flight of stairs at the time of study enrollment[23], [24]. The others were drawn from the general Memphis population ascertained by self-referral in response to advertising and presentations at community establishments for the elderly. Because differences in allele frequencies between ethnic groups have been reported for some of the genetic variants used in our models,[20] and there was an insufficient number of blacks available in the VM dataset on which to build a model (n = 3 with complete risk factor data), blacks in the ARMA cohort were not included in any analyses.

Large variation in the demographic characteristics of the training and testing datasets, though informative about how well these models may apply to a general population, will reduce the number of individuals correctly classified in the testing dataset. Therefore, we carefully compared the testing and training datasets to determine if they differed substantially for any important demographic traits (Table 2).

**Table 2:** Characteristics of the datasets.

Characteristic	VM Training	VM Testing	VM Families	ARMA	p-value VM Training vs. ARMA
Cases/Affecteds (#)	349	87	326	85	NA
Controls/Unaffecteds (#)	216	54	86	148	NA
Age of exam [mean (sd)]	73.5 (8.4)	73.1 (8.3)	72.8 (9.4)	79.3 (3.6)	<0.0001
Gender (% Female)	61.1	59.6	67.0	51.5	0.01
% ever Smokers	52.0	56.0	54.6	50.2	0.64
CFH frequency C allele	50.6	48.9	61.9	42.9	0.01
ARMS2 frequency T allele	35.7	31.2	42.4	23.8	<0.0001
CFB frequency A allele	6.8	7.1	5.2	9.7	0.05
C3 frequency C allele	25.3	26.2	29	25.5	0.93

### Genotyping

CFH Y402H (rs1061170), ARMS2 A69S (rs10490924), CFB R32Q (rs641153), and C3 R102G (rs2230199) were genotyped as part of a Sequenom iPLEX® Goldpool, according to the manufacturer's instructions. Three quality control samples were duplicated within and between plates, and genotypes were checked for concordance. All genetic variants had a genotyping efficiency rate of at least 95%. Because the algorithm requires complete risk factor information, the few samples that did not have complete genotype information were dropped from analysis. All SNPs were verified to be in Hardy-Weinberg equilibrium in controls.

### Building the Models

Logistic regression, MDR, and GENN were used to build models of AMD. For the logistic regression analyses, we included age of examination (in years), smoking (coded “1” for those who had smoked at least 100 cigarettes, “0” for those who reported never smoking or smoking less than 100 cigarettes), and CFH Y402H, ARMS2 A69S, CFB R32Q, and C3 R102G (using additive encodings for all genetic variants) in the model. Though other environmental variables (e.g. sex and body mass index (BMI)[25]) and genetic variants (e.g. polymorphisms in or near the CFI[26], LIPC[27], and TIMP3[28] genes) may also be associated with AMD, we chose not to include them in the model, primarily for two reasons: 1) to minimize the number of parameters the model estimated we chose only the most robustly associated genetic and environmental factors with the greatest effect sizes, and 2) some of the modeling methods we wanted to test (e.g. MDR) perform optimally with categorical variables, rather than quantitative traits like BMI. Therefore, the logistic regression equation was:
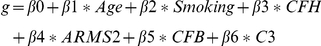
and a rough estimate of the probability of AMD for an individual can be calculated as:
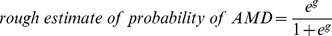
Based on the size of the available datasets, we did not include interactions terms in the model, thereby reducing the number of parameters that have to be estimated. Once the rough estimate of probability of AMD was determined for each individual in the testing dataset, individuals with a probability greater than a particular threshold were classified as “high-risk”, and those below the threshold were classified as “low-risk”. These “model calls” were then compared to the affection status assigned by a clinician, and the sensitivity, specificity, positive predictive value, negative predictive value, and overall correct classification rate of the model were determined. Changing the threshold for the probability of AMD will change the number of false positives and false negatives called by the model. Because there was no a priori reason to select a particular threshold value, we chose 0.5 as a cut-off for our analyses. After examining the histogram of AMD probabilities by true affection status, we raised the threshold to 0.75 in the ARMA testing dataset in an attempt to increase accuracy. Finally, we used ROC curves (plots of sensitivity vs. 1-specificity) to determine the threshold that would have correctly classified the greatest number of individuals.

For MDR, the number of cases and controls with each particular susceptibility factor combination was calculated. If the ratio of cases to controls having this combination in the training dataset exceeded the total ratio of cases and controls, then individuals with the same combination in the testing dataset were called “high-risk”. Otherwise, individuals were called “low-risk”. This is the usual metric used to classify individuals as “high-” or “low-risk” by the MDR method[29], and MDR software[30] was used to generate the counts of cases and controls with each combination of susceptibility factors in the training dataset. However, this does not exactly correspond to a “traditional” MDR analysis because: 1) we a priori forced MDR to include our variables of interest, rather than allowing the software to perform variable selection and 2) we used completely independent datasets for testing rather than cross validation. We included smoking and the CFH, ARMS2, CFB, and C3 variants in the model. Because MDR works best with susceptibility factors that have only a few levels, and because we wanted to maintain comparability with the logistic regression analyses, age of exam was included in the model coded “1” for individuals in the lowest quantile of age of exam, “2” for those in the second quantile, and so on. One advantage of this type of MDR model compared to logistic regression is that there is no need to specify an arbitrary threshold value for classifying risk status. The major drawbacks are: 1) age cannot be included in the model as a continuous variable without overly stratifying the datasets and 2) large sample sizes are needed for each susceptibility combination to ensure stability of the model.

Grammatical Evolution of Neural Networks (GENN) has been extensively described[31]. Briefly, neural networks are a robust and flexible modeling strategy, consisting of input layers, hidden layers, and an output layer. Each layer contains various nodes connected by arcs and weighted by some arithmetic function. When the input data exceed some threshold, the neural network “fires”. The goal in our case was to classify individuals as high- or low-risk for AMD (the output) from genetic and clinical risk factor data (the inputs). The architecture of the neural network (how the nodes are connected, the weights on each node, etc.) was optimized using the process of grammatical evolution. Grammatical evolution begins with an initial random set of neural network architectures, and the neural networks with the best fitness (measured in this application by balanced accuracy[32]) are propagated to the next generation. Random “mutation” and “crossover” events in subsequent generations allow the neural networks to evolve, and hopefully, reach a final architecture that is useful in classifying AMD risk level. To maintain comparability with the other modeling strategies used, we included age of exam (in quantiles), smoking, and CFH Y402H, ARMS2 A69S, CFB R32Q, and C3 R102G as inputs for the neural networks. Only neural networks that contained all 6 factors exactly once were propagated to the next generation (effectively allowing for optimization of the weights and arcs, but not allowing variable selection). The parameter settings for the evolution of the neural networks were a “genome size” ranging from 25–1000 bits, using a population size of 5000 “genomes”, with probability of a crossover event set to 0.9, and probability of mutation set to 0.01. After optimization of the neural networks in the training dataset, the final best neural network model, as measured by balanced accuracy in the training dataset, was applied to the testing dataset and evaluated.

### Evaluating the Models

We used AMD case-control status assigned by retinal specialists after examination of stereoscopic fundus photographs as the gold standard with which to compare our “model calls” of high- and low-risk from the three methods. Though the possibility of clinician misdiagnosis exists, extensive quality control measures were taken to guard against it, including concordance checks by multiple graders. In a previous study using a subset of the VM datasets, concordance among graders was 92% with a kappa statistic of 0.81, indicating excellent agreement[33].

We defined sensitivity as the number of individuals who were truly affected with AMD and identified by the algorithm as “high-risk”/total number of true AMD cases as determined by clinician grading. Specificity equals the number of individuals who were truly unaffected and called “low-risk”/total number of controls. The overall correct classification rate is the number of true cases identified as “high-risk” plus the number of controls identified as “low-risk”/total number of individuals tested. PPV equals the percentage of individuals labeled “high-risk” who were true AMD cases. NPV equals the percentage of individuals labeled “low-risk” who were true controls. Estimates of PPV and NPV from case-control data are often inflated[34], and can be adjusted by considering the prevalence of the disease in the population of interest using the following formulas:
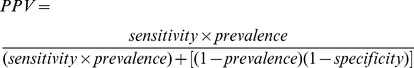


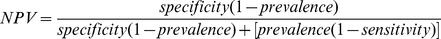
As the ARMA cohort is primarily population-based, we report only the unadjusted PPV and NPV in this dataset.

## Results

### Model Building in the VM Training Dataset

In the logistic regression model built in the VM training dataset, all six susceptibility factors were significantly associated with AMD in the direction expected from previous reports in the literature (Table 3). In the MDR model, there was a clear tendency for individuals with more risk alleles/risk factors present and fewer protective alleles to be classified by the model as “high-risk” and vice versa, as expected (data not shown). GENN separated the genetic risk factors and the environmental risk factors into two separate hubs (Figure S1). Of the genetic factors, GENN weighted CFH Y402H and ARMS2 A69S most heavily, followed by C3 R102G and CFB R32Q. This ranking of the genetic susceptibility factors mimics the frequency of these factors in our training dataset. However, it is difficult to infer the relative importance of each factor to disease from the GENN weights alone because the weights will change with tweaks to the architecture of the neural network.

**Table 3:** Logistic regression model in the VM training dataset.

Factor	Coefficient	p-value	Odds Ratio	95% Confidence Interval	
Age	0.13	<0.001	1.13	1.10	1.17
Smoking	0.48	0.026	1.61	1.06	2.45
CFH Y402H	1.04	<0.001	2.84	2.07	3.90
ARMS2 A69S	0.69	<0.001	2.00	1.47	2.72
CFB R32Q	−1.10	<0.001	0.33	0.18	0.60
C3 R102G	0.41	0.014	1.51	1.09	2.11
Constant	−10.48	<0.001	.	.	.

### Models Built in the VM Training Dataset Applied to VM Testing and VM Family Datasets

Of the three methods, the GENN model performed the best when applied to the VM testing dataset with an overall correct classification rate of 80.1% (Table 4). GENN correctly classified more cases than controls (83.9% sensitivity vs. 74.1% specificity). The logistic regression model was slightly less successful than GENN (77.3% overall correct), and followed the same trend of higher classification rates for cases than controls. The area under the ROC curve was 0.84 (95% confidence interval 0.81 to 0.88, Figure S2), which exceeded both the AUC for previously developed similar algorithms (Table 1) and the recommended cutoff for screening high-risk individuals[19].

**Table 4:** Classification rates using the VM training dataset for training and VM testing dataset for testing.

Method	Sensitivity	Specificity	Unadjusted PPV	Unadjusted NPV	% Overall Correct
LR [0.5]	85.1	64.8	79.6	72.9	77.3
MDR	71.8 (58.6)	80.5 (61.1)	86.4 (NA)	62.3 (NA)	75.0 (59.6)
GENN	83.9	74.1	83.9	74.1	80.1
Consensus–LR, MDR, GENN	82.8	74.1	83.7	72.7	79.4
Consensus–LR, GENN	77.0	74.1	82.7	66.7	75.9

PPV = positive predictive value, NPV = negative predictive value, NA = not applicable, LR = logistic regression. Logistic [0.5] indicates the threshold used for determining model calls in the logistic regression analysis. In this case, all individuals with probabilities ≥0.5 were given a model call of “high-risk”. For MDR 20.6% of the testing dataset could not be classified. The first entry in the table represents the classification rate considering only the individuals that could be classified in the denominator. The number in parentheses gives the classification rate considering the entire testing dataset as the denominator. For example, using MDR, 71 individuals who were actually cases could be classified and of those 51/71 = 71.8% were correctly classified as “high-risk”. Considering all cases that were tested, 51/87 = 58.6% were correctly classified. For the consensus of methods, individuals were called high-risk only if two or more methods classified them as high-risk.

The MDR method did much worse than GENN and logistic regression with only 59.6% of individuals classified correctly and 20.6% of the individuals not classified at all (denoted “CNC” for could not classify). When a particular combination of susceptibility factors is not observed in the training dataset, no decision rule can be made, and therefore all individuals with that combination in the testing dataset are CNC. Given the sample size of the available datasets and the number of factors in the model, the somewhat decreased performance of the MDR model is not unexpected.

We also examined the agreement between the three methods using a consensus approach that gives individuals a “high-risk” call only when at least 2 of the 3 methods indicate increased risk and a “low-risk” call otherwise. Taking the consensus of all three methods or of the two best-performing individual methods (logistic regression and GENN) classified fewer people correctly than using GENN alone and did not improve either the sensitivity or specificity.

Naïve estimates of PPV and NPV are known to be inaccurate when calculated from case-control data[34]. We compared the naïve estimates to adjusted estimates using prevalence rates of 5.5% and 15% (Table 5). As expected, the PPV for each method decreased, ranging from 12.3 to 17.6 at a prevalence of 5.5% and from 29.9 to 39.4% at a prevalence of 15%. Notably, the NPV increased substantially, exceeding 94% for all methods tested at both prevalence rates.

**Table 5:** Comparison of adjusted and unadjusted PPV and NPV in the VM testing dataset.

Method	Unadjusted PPV	Unadjusted NPV	Adjusted PPV at Prev = 5.5%	Adjusted NPV at Prev = 5.5%	Adjusted PPV at Prev = 15%	Adjusted NPV at Prev = 15%
LR 0.5	79.6	72.9	12.3	98.7	29.9	96.1
MDR	86.4	62.3	17.6	98.0	39.4	94.2
GENN	83.9	74.1	15.9	98.8	36.4	96.3
Consensus–LR, MDR, GENN	83.7	72.7	15.7	98.7	36.1	96.1
Consensus–LR, GENN	82.7	66.7	14.8	98.2	34.4	94.8

Prev =  Prevalence.

We next applied the same model to a testing dataset composed of families with multiple members affected by AMD. In the family data, logistic regression performed best overall, but only by a small margin over GENN (overall correct classification rates of 76.9% and 73.3%, respectively, Table S1). Logistic regression was more sensitive than GENN, but less specific (sensitivity 84.0% vs. 76.1%, specificity 50.0% vs. 62.8%, respectively). MDR again had the poorest accuracy of the three methods (overall correct classification 71.4%) with a similar proportion of individuals called CNC (17.7%). Taking the consensus of logistic regression and GENN improved the specificity compared to each single method alone (69.8%), at the expense of lower sensitivity than either single method (74.5%).

### Models Built in the VM Training Dataset Applied to the ARMA Dataset

For a more realistic measure of how these models apply to the general population, we tested them in the ARMA dataset. As expected, the classification rates did decrease, but were still better than chance. Because the optimal threshold for the probability cutoff in logistic regression is likely to vary by dataset and this threshold cannot be determined in advance, we examined three cut-offs: 1) 0.5, chosen for comparison with the VM testing dataset analysis 2) 0.75, chosen after examining a histogram of probabilities by clinician-assigned affection status (data not shown), and 3) 0.87, chosen because this was the optimal threshold in the ARMA dataset determined by ROC analysis (Figure S3). Using the optimal 0.87 threshold, 69.1% of individuals were correctly classified yielding a sensitivity of 36.5% and specificity of 87.8% (Table 6). Obviously, decreasing the threshold resulted in suboptimal classification rates: 60.5% overall correct for a threshold of 0.75 and 48.9%, worse than chance, for a threshold of 0.5. This suggests that if the logistic model were to be applied to a new population, a sample of that population would need to be tested and the model carefully calibrated before widespread use. Model calibration strategies have been successfully used to adjust an algorithm for coronary heart disease that was created in the Framingham Heart Study for application in six other ethnically diverse cohorts[35], and we expect that using a similar approach would increase the accuracy of our algorithm in other populations.

**Table 6:** Classification rates using the VM training dataset for training and the ARMA dataset for testing.

Method	Sensitivity	Specificity	Unadjusted PPV	Unadjusted NPV	% Overall Correct
LR [0.5]	89.4	25.7	40.9	80.9	48.9
LR [0.75]	62.4	59.5	46.9	73.3	60.5
LR [0.87, Optimal)	36.5	87.8	63.3	70.7	69.1
MDR	68.5 (58.8)	31.4 (25.0)	38.2 (NA)	61.7 (NA)	45.5 (37.3)
GENN	76.5	36.5	43.6	73.0	51.1
Consensus–LR [0.5], MDR, GENN	77.6	33.8	40.2	72.5	49.8
Consensus–LR [0.5], GENN	74.1	41.9	42.3	73.8	53.6
Consensus–LR [0.75], MDR, GENN	64.7	53.4	44.4	72.5	57.5
Consensus–LR [0.75], GENN	61.2	59.5	46.4	72.7	60.1
Consensus–LR [0.87], MDR, GENN	60.0	58.1	45.1	71.7	58.8
Consensus–LR [0.87], GENN	36.5	87.8	63.3	70.7	69.1

Logistic [0.87 Optimal] indicates that the threshold that would correctly classify the most individuals as determined by the ROC curve was applied to the testing dataset.

See notes accompanying Table 4 for further explanation.

Somewhat surprisingly, the GENN model, which performed the best in the VM testing dataset, was not as successful in the ARMA dataset (51.1% overall correct). Though quite good at identifying cases (76.5% sensitivity), the method was hampered by poor performance in controls (36.5% specificity). MDR was again the worst with an overall classification rate of 45.5%, and leaving 18.0% of the data unclassified.

Taking the consensus of logistic regression with the optimal threshold and GENN resulted in the same sensitivity, specificity, and overall classification rate as using logistic regression with the optimal threshold alone (Table 6), as everyone called high-risk by logistic regression was also labeled high-risk by GENN. Interestingly, if model calibration cannot be performed and we must use the arbitrary threshold of 0.5 for logistic regression, then taking the consensus of logistic regression and GENN is more successful than either method individually.

As a final check of how well the models would apply in population-based data, we removed all individuals ascertained in the ARMA cohort who were not part of the Health ABC Study. This produced very similar results for all methods (Table S2). Logistic regression at the optimal threshold again had the highest overall classification rate (70.4%). Assuming no prior knowledge of the optimal threshold for logistic regression, the most successful algorithm was again taking the consensus of logistic regression at a threshold of 0.5 and GENN.

### Comparison of VM Training Dataset to the ARMA Dataset

The VM training and ARMA datasets were ascertained using very different strategies. The VM training dataset was drawn from ophthalmology, primarily retinal, clinics. The ARMA dataset was primarily drawn from the Memphis Health ABC cohort, which randomly sampled those on Medicare rolls. The VM training dataset had a higher percentage of females, a higher frequency of CFH and ARMS2 risk alleles, and a lower percentage of CFB protective alleles (Table 2), as might be expected when comparing a clinic-based group to the general population. However, though these differences are not unexpected, they still negatively affect performance of all three methods, and partially explain the decreased accuracy observed in the ARMA dataset.

### Model Building in the ARMA Dataset

Next, we rebuilt the model using the ARMA dataset. Unfortunately, with 85 cases, most of whom were AREDS category 3 (i.e., not advanced AMD), and 148 controls, the ARMA dataset was somewhat underpowered to detect significant effects of all the established AMD susceptibility factors we studied. This was especially apparent in the logistic regression analysis, where only CFH Y402H and CFB R32Q were significantly associated with AMD risk (Table 7). The sparseness of data would also be expected to have a detrimental effect on the other 2 methods, especially MDR, which depends on large numbers of observations for each combination of susceptibility factors to ensure stability of the model. Nonetheless, we still observed a clear trend for those carrying more risk factors and fewer protective CFB alleles to be called “high-risk” by MDR. The neural network model was remarkably similar to the model produced in the VM training dataset, with the network again containing separate hubs for genetic and environmental susceptibility factors, and the same ranking of weights given to the genetic risk factors (Figure S4).

**Table 7:** Logistic regression model in the ARMA dataset.

Factor	Coefficient	p-value	Odds Ratio	95% Confidence Interval	
Age	0.05	0.22	1.05	0.97	1.14
Smoking	0.41	0.16	1.51	0.85	2.68
CFH Y402H	0.73	<0.0001	2.08	1.39	3.11
CFB R32Q	−0.96	0.02	0.38	0.17	0.86
ARMS2 A69S	0.37	0.12	1.45	0.91	2.31
C3 R102G	−0.03	0.89	0.97	0.61	1.53
Constant	−5.45	0.10	.	.	.

### Models Built in the ARMA Dataset Applied to the VM Datasets

Since the VM training and testing datasets were created by randomly assigning 4/5 of individuals for training and 1/5 for testing, we combined them for the purposes of creating a testing dataset for models built in the ARMA dataset. Using a probability threshold of 0.5, logistic regression correctly classified 59.3% of individuals. Decreasing the threshold drastically improved performance. Using the optimal threshold of 0.30 determined by the ROC curve (Figure S5), 76.2% of individuals were correctly classified (79.6% sensitivity, 70.7% specificity, Table 8). GENN performed better than logistic regression using the arbitrary 0.5 threshold with 63.0% of individuals correctly classified, but was not as successful as logistic regression using the optimal threshold. MDR performed poorly, leaving 57.2% of the data unclassified, and only correctly classifying 49.0% of individuals who were given a result. Taking the consensus of logistic regression and GENN did not provide a better overall classification rate than using logistic regression with the optimal threshold alone, but did increase the specificity (79.6%) at the cost of lowering sensitivity (61.2%).

**Table 8:** Classification rates using the ARMA dataset for training and VM training combined with VM testing as the testing dataset.

Method	Sensitivity	Specificity	Unadjusted PPV	Unadjusted NPV	% Overall Correct
LR [0.5]	37.4	94.8	92.1	48.4	59.3
LR [0.30 Optimal]	79.6	70.7	81.5	68.2	76.2
MDR	48.8 (24.1)	49.4 (15.9)	70.5 (NA)	28.1 (NA)	49.0 (21.0)
GENN	65.4	59.3	72.2	51.4	63.0
Consensus–LR [0.5], MDR, GENN	42.7	90.0	87.3	49.3	60.8
Consensus–LR [0.5], GENN	35.6	94.8	91.7	47.7	58.2
Consensus–LR [0.3], MDR, GENN	65.4	75.9	81.4	57.6	69.4
Consensus–LR [0.3], GENN	61.2	79.6	82.9	56	68.3

For MDR 57.2% of the testing dataset could not be classified. The first entry in the table represents the classification rate considering only the individuals that could be classified in the denominator. The number in parentheses gives the classification rate considering the entire testing dataset as the denominator.

## Discussion

Many strategies have been used by our group and others to identify individuals at elevated risk for AMD. Whenapplying a model to a new dataset, we have seen that taking the consensus of logistic regression and GENN models maximizes the overall classification rate compared to any single method, when the optimal threshold for logistic regression is not known. Using this approach we classified individuals into “high-“ or “low-risk” groups, with overall correct classification rates of ∼76% in the VM testing data and nearly 70% in the ARMA cohort. These numbers are impressive, but PPV (the percentage of individuals labeled “high-risk” who are actually cases) and NPV (the percentage of individuals labeled “low-risk” who are actually controls) are generally more informative when considering the potential clinical usefulness of a new algorithm. Because of the difficulty in accurately estimating PPV and NPV in case-control data, it is essential to validate models in population-based cohorts. In the ARMA cohort, using logistic regression alone produced identical PPV and NPV results (∼63% and ∼71%, respectively) as taking the consensus of logistic regression and GENN models when the optimal threshold for logistic regression was used. Remarkably, even using the arbitrary 0.5 threshold for logistic regression, when taking the consensus with GENN, the PPV (∼42%) and NPV (∼74%) were still quite high.

To put our results in perspective, we compared them to three other screening tools commonly used in clinical practice: the prostate-specific antigen (PSA) and digital rectal exam (DRE) for prostate cancer and mammography for breast cancer. A recent meta-analysis estimated the sensitivity, specificity, and PPV for PSA at 72.1%, 93.2%, and 25.1%, respectively and at 53.2%, 83.6%, and 17.8% for DRE[36]. Practically speaking, this means that out of all individuals with an abnormal PSA or DRE result, only about 1 in 4 or 5 actually has prostate cancer. When the PSA and DRE are normal, ∼90% are cancer-free[36]. Despite the low PPV, the American Cancer Society recommendsthat physicians counsel men over 50who are expected to live at least 10 years about the benefits and risks of PSA tests and DRE and begin counseling at age 40–45 for men in certain high-risk groups[37].

In a review conducted for the U.S. Preventive Services Task Force, first mammography sensitivity ranged from 71–96%, specificity for a single mammographic exam ranged from 94–97%, and the PPV ranged from 2–22% for abnormal results that led to further evaluation and 12–78% for abnormal results leading to biopsy[38]. Again the PPV values are surprisingly low, but the justification for regular mammograms is bolstered by additional studies demonstrating lower mortality rates from breast cancer among women who undergo regular screening. Comparable prospective studies in AMD would need to show that being able to identify high-risk individuals leads to better visual outcomes before widespread screening would be recommended. Such studies, to our knowledge, have not been conducted for any of the AMD algorithms described in the literature.

Ultimately, the decision to use a particular algorithm in clinical practice is a judgment call that must balance the need to flag all potentially high-risk persons with the cost of falsely labeling some low-risk individuals as high-risk. Extensive clinical validation studies, in particular applying potential algorithms to prospective cohorts, need to be implemented. Furthermore, many other factors besides clinical validity also deserve significant attention, including the cost of screening and what can be done to help those who are classified as high-risk to prevent disease. Though we believe that it is premature to introduce such an AMD algorithm to the clinic now, these results demonstrate promise for the potential of genetic variants in predicting individual risk for disease.

## Supporting Information

Figure S1 **Neural network model developed in the VM training dataset.** W = weight, PADD = addition function.
(DOCX)

Figure S2 **ROC analysis in the VM training dataset.** Area under the ROC  = 0.84 (95% confidence interval 0.81 to 0.88)
(DOCX)

Figure S3 **ROC analysis in the ARMA dataset.** Area under the ROC  = 0.67 (95% confidence interval 0.59 to 0.74.)
(DOCX)

Figure S4 **Neural network model developed in the ARMA dataset.** W = weight, PADD = addition function.
(DOCX)

Figure S5 **ROC analysis in the combined VM training and VM testing dataset.** Area under the ROC  = 0.82 (95% confidence interval 0.79 to 0.85)
(DOCX)

Table S1 **Classification rates using the VM training dataset for training and the VM family dataset for testing.**
(DOCX)

Table S2 **Classification rates using the VM training dataset for training and only Health ABC individuals from ARMA for testing.**
(DOCX)
