# Supplementary material for: Using Genetic Variation and Environmental Risk Factor Data to Identify Individuals at High Risk for Age-Related Macular Degeneration
Source: PLoS One. 2011 Mar 24;6(3):e17784. doi: 10.1371/journal.pone.0017784 (PMC3063776; doi:10.1371/journal.pone.0017784)

Supplementary Figure 1. Neural Network Model Developed in the VM Training Dataset

W=weight, PADD=addition function.


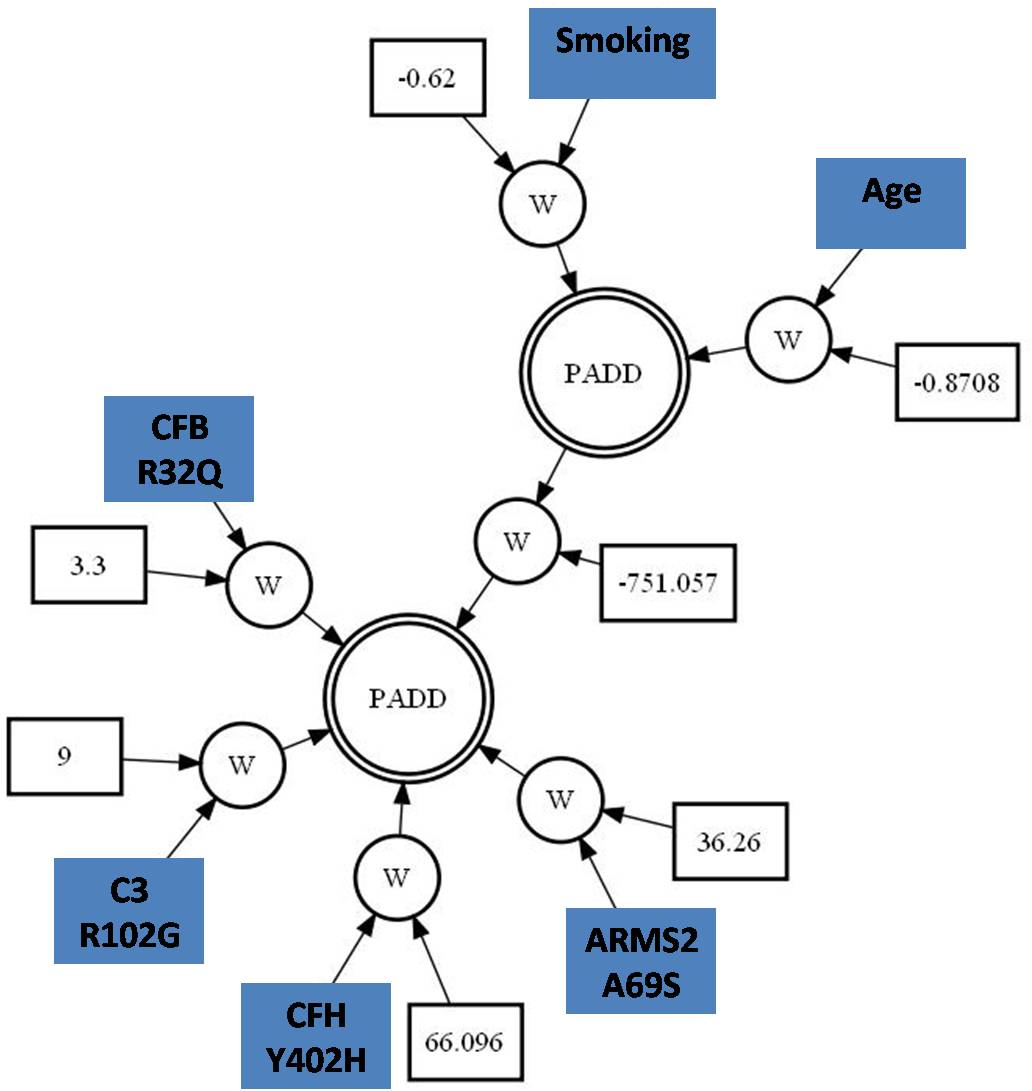

Supplement: Figure S1 — Neural network model developed in the VM training dataset. W = weight, PADD = addition function. (DOCX) [file pone.0017784.s001.docx]
