# Supplementary material for: Using Genetic Variation and Environmental Risk Factor Data to Identify Individuals at High Risk for Age-Related Macular Degeneration
Source: PLoS One. 2011 Mar 24;6(3):e17784. doi: 10.1371/journal.pone.0017784 (PMC3063776; doi:10.1371/journal.pone.0017784)

Supplementary Figure 2. ROC Analysis in the VM Training Dataset

Area under the ROC = 0.84 (95% confidence interval 0.81 to 0.88)


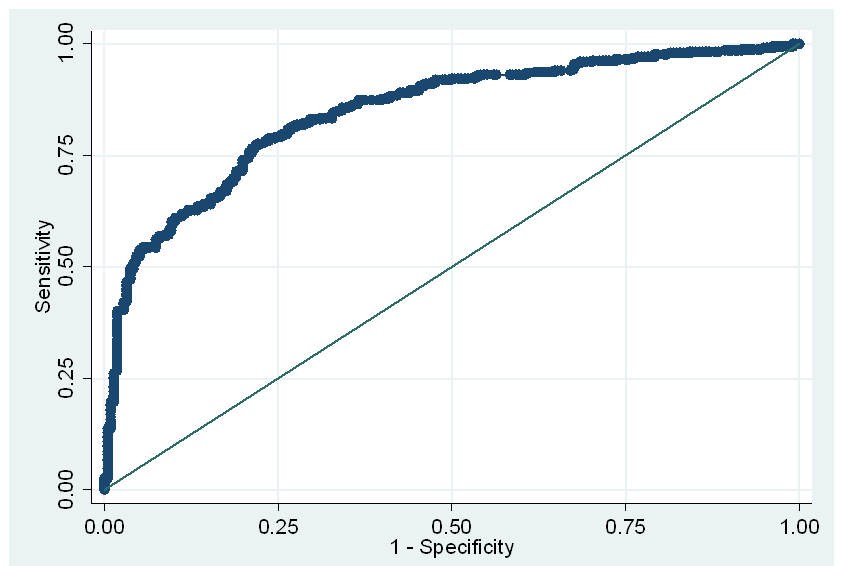

Supplement: Figure S2 — ROC analysis in the VM training dataset. Area under the ROC = 0.84 (95% confidence interval 0.81 to 0.88) (DOCX) [file pone.0017784.s002.docx]
