# Supplementary material for: Using Genetic Variation and Environmental Risk Factor Data to Identify Individuals at High Risk for Age-Related Macular Degeneration
Source: PLoS One. 2011 Mar 24;6(3):e17784. doi: 10.1371/journal.pone.0017784 (PMC3063776; doi:10.1371/journal.pone.0017784)

Supplementary Figure 3. ROC Analysis in the ARMA Dataset

Area under the ROC = 0.67 (95% confidence interval 0.59 to 0.74)


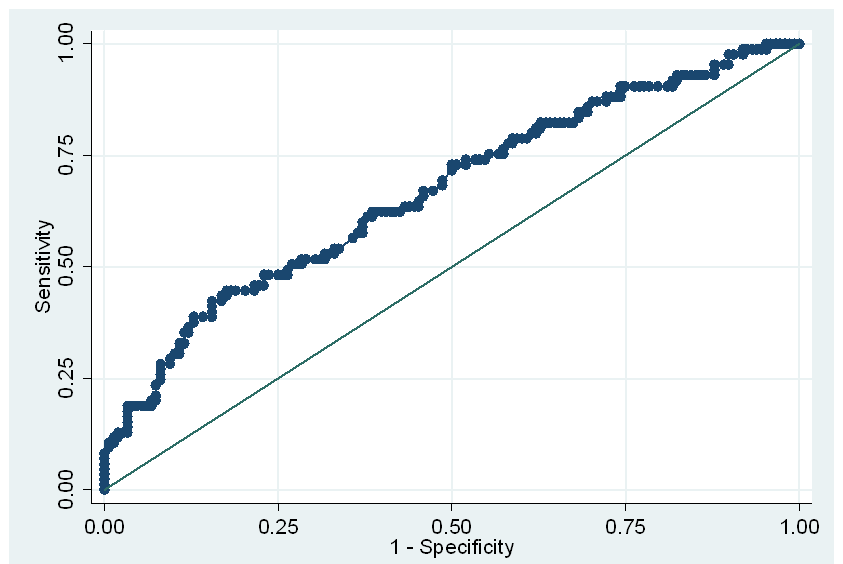

Supplement: Figure S3 — ROC analysis in the ARMA dataset. Area under the ROC = 0.67 (95% confidence interval 0.59 to 0.74.) (DOCX) [file pone.0017784.s003.docx]
