# Supplementary material for: Using Genetic Variation and Environmental Risk Factor Data to Identify Individuals at High Risk for Age-Related Macular Degeneration
Source: PLoS One. 2011 Mar 24;6(3):e17784. doi: 10.1371/journal.pone.0017784 (PMC3063776; doi:10.1371/journal.pone.0017784)

Supplementary Figure 5. ROC Analysis in the Combined VM training and VM Testing Dataset

Area under the ROC = 0.82 (95% confidence interval 0.79 to 0.85)


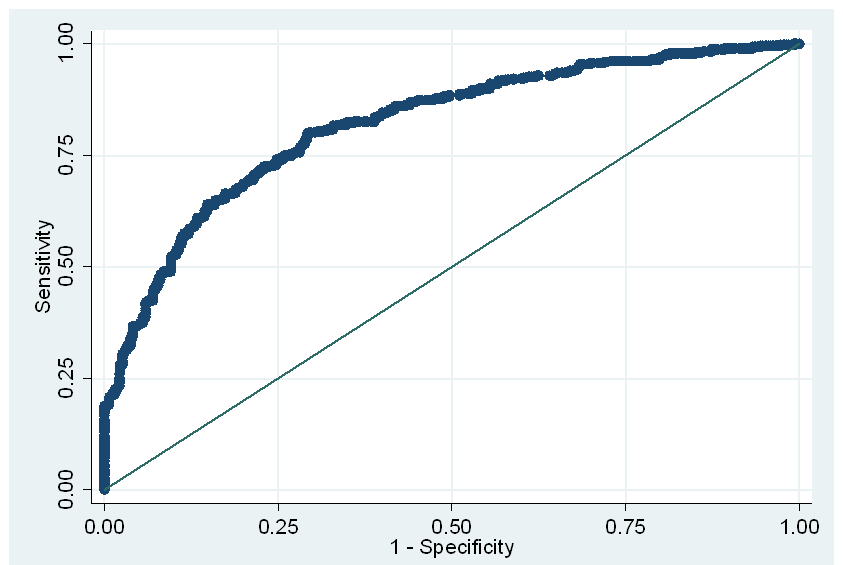

Supplement: Figure S5 — ROC analysis in the combined VM training and VM testing dataset. Area under the ROC = 0.82 (95% confidence interval 0.79 to 0.85) (DOCX) [file pone.0017784.s005.docx]
