# Supplementary material for: Using Genetic Variation and Environmental Risk Factor Data to Identify Individuals at High Risk for Age-Related Macular Degeneration
Source: PLoS One. 2011 Mar 24;6(3):e17784. doi: 10.1371/journal.pone.0017784 (PMC3063776; doi:10.1371/journal.pone.0017784)
Supplement: Table S1 — Classification rates using the VM training dataset for training and the VM family dataset for testing. (DOCX) [file pone.0017784.s006.docx]

Supplementary Table 1. Classification Rates Using the VM Training Dataset for Training and the VM Family Dataset for Testing

| **Method** | **Sensitivity** | **Specificity** | **Unadjusted PPV** | **Unadjusted NPV** | **% Overall Correct** |
| --- | --- | --- | --- | --- | --- |
| LR [0.5] | 84.0 | 50.0 | 86.4 | 45.3 | 76.9 |
| MDR | 73.7 (60.1) | 63.0 (53.5) | 87.9 (NA) | 39.7 (NA) | 71.4 (58.7) |
| GENN | 76.1 | 62.8 | 88.6 | 40.9 | 73.3 |
| Consensus--LR [0.5], MDR, GENN | 77.3 | 59.3 | 87.8 | 40.8 | 73.5 |
| Consensus--LR [0.5], GENN | 74.5 | 69.8 | 90.3 | 42.0 | 73.5 |
