# Supplementary material for: Using Genetic Variation and Environmental Risk Factor Data to Identify Individuals at High Risk for Age-Related Macular Degeneration
Source: PLoS One. 2011 Mar 24;6(3):e17784. doi: 10.1371/journal.pone.0017784 (PMC3063776; doi:10.1371/journal.pone.0017784)
Supplement: Table S2 — Classification rates using the VM training dataset for training and only Health ABC individuals from ARMA for testing. (DOCX) [file pone.0017784.s007.docx]

Supplementary Table 2. Classification Rates Using the VM Training Dataset for Training and Only Health ABC Individuals from ARMA for Testing

| **Method** | **Sensitivity** | **Specificity** | **Unadjusted PPV** | **Unadjusted NPV** | **% Overall Correct** |
| --- | --- | --- | --- | --- | --- |
| LR [0.5] | 90.8 | 22.4 | 36.2 | 83.3 | 44.7 |
| LR [0.75] | 66.2 | 56.7 | 42.6 | 77.6 | 59.8 |
| LR [0.87, Optimal) | 36.9 | 86.6 | 57.1 | 73.9 | 70.4 |
| MDR | 70.4 (58.5) | 27.4 (21.6) | 33 (NA) | 64.4 (NA) | 41.9 (33.7) |
| GENN | 80.0 | 31.3 | 36.1 | 76.4 | 47.2 |
| Consensus--LR [0.5], MDR, GENN | 80.0 | 29.1 | 35.4 | 75.0 | 45.7 |
| Consensus--LR [0.5], GENN | 76.9 | 37.3 | 37.3 | 76.9 | 50.3 |
| Consensus--LR [0.75], MDR, GENN | 67.7 | 50.0 | 39.6 | 76.1 | 55.8 |
| Consensus--LR [0.75], GENN | 64.6 | 56.7 | 42.0 | 76.8 | 59.3 |
| Consensus--LR [0.87], MDR, GENN | 61.5 | 55.2 | 40.0 | 74.7 | 57.3 |
| Consensus--LR [0.87], GENN | 36.9 | 86.6 | 57.1 | 73.9 | 70.4 |
